# Cardiac Magnetic Resonance Image Segmentation Method Based on Multi-Scale Feature Fusion and Sequence Relationship Learning

**DOI:** 10.3390/s23020690

**Published:** 2023-01-07

**Authors:** Yushi Qi, Chunhu Hu, Liling Zuo, Bo Yang, Youlong Lv

**Affiliations:** 1 College of Mechanical Engineering, Donghua University, Shanghai 201620, China; 2 Institute of Artificial Intelligence, Donghua University, Shanghai 201620, China

**Keywords:** CNN, RNN, cardiac MRI, multi-scale, dual attention, sequence relationship

## Abstract

Accurate segmentation of the left atrial structure using magnetic resonance images provides an important basis for the diagnosis of atrial fibrillation (AF) and its treatment using robotic surgery. In this study, an image segmentation method based on sequence relationship learning and multi-scale feature fusion is proposed for 3D to 2D sequence conversion in cardiac magnetic resonance images and the varying scales of left atrial structures within different slices. Firstly, a convolutional neural network layer with an attention module was designed to extract and fuse contextual information at different scales in the image, to strengthen the target features using the correlation between features in different regions within the image, and to improve the network’s ability to distinguish the left atrial structure. Secondly, a recurrent neural network layer oriented to two-dimensional images was designed to capture the correlation of left atrial structures in adjacent slices by simulating the continuous relationship between sequential image slices. Finally, a combined loss function was constructed to reduce the effect of positive and negative sample imbalance and improve model stability. The Dice, IoU, and Hausdorff distance values reached 90.73%, 89.37%, and 4.803 mm, respectively, based on the LASC2013 (left atrial segmentation challenge in 2013) dataset; the corresponding values reached 92.05%, 89.41% and 9.056 mm, respectively, based on the ASC2018 (atrial segmentation challenge at 2018) dataset.

## 1. Introduction

Atrial fibrillation (AF) is one of the most common arrhythmic conditions and is prone to a variety of complications, such as thrombosis and heart failure. The occurrence of AF is closely associated with fibrosis of the left atrium, and segmentation of the patient’s left atrial structure from cardiac magnetic resonance images for analysis and study is an important basis for the diagnosis and treatment of AF using robotic surgery [1]. In robotic surgery, computer-assisted image segmentation techniques [2] have been widely used because they can avoid the dependence of manual image segmentation techniques on the surgeon’s personal experience and can thereby effectively reduce bias [3]. However, cardiac MRI images are 3D sequential images, in which a set of images usually contains dozens or even hundreds of slices, and the size and shape of the left atrium in each slice are different, so segmenting a set of images involves a large and time-consuming workload. In addition, MRI images contain multiple cardiac tissue structures, and the contrast between different tissue structures is low, which means that the segmenter must possess a high level of expertise [4]. The above features increase the difficulty of cardiac MRI image segmentation.

Traditional image segmentation algorithms mainly include the threshold segmentation method [5], region growing algorithm [6], clustering-based [7] methods, etc. Gomez et al. [8] also provided an extensive list of methods in the 2013 left atrial segmentation challenge, all of which have yielded excellent results in the left atrial segmentation problem, with some of the metrics still leading the way today. With the development of medical technology, different types and qualities of cardiac magnetic resonance images pose a challenge for the accurate segmentation of left atrial structures [9]. With the rapid development of deep learning [10], an increasing number of scholars have begun to exploit the advantages of deep learning methods with automatic feature extraction, strong learning ability, and easy training to meet the requirement of accurate segmentation of medical images [11]. Yashu Liu et al. [12] designed two image segmentation networks based on the FCN structure and U-Net structure and implemented left atrial structure segmentation by improving the Dice loss function to reduce the effect of imbalance between positive and negative samples. Zhaohan Xiong et al. [13] proposed an FCN-based automatic left atrial segmentation method to improve the segmentation accuracy of left atrial structural details by designing a dual-pathway architecture to capture the local geometry and global position information of the left atrium. Sulaiman Vesal et al. [14] proposed a 3D fully convolutional automatic segmentation network based on 3D U-Net [15], which uses dilated convolution at the bottom layer of the network and introduces residual connections to integrate local and global information to improve the segmentation accuracy, but this method requires a large dataset [16] and has difficulty capturing long-range dependencies [17]. Mourad Gridach proposed PyDiNet [18] to improve the segmentation accuracy of image details by capturing small and complex changes in medical images while preserving global information with the dilation convolution of the pyramid structure. Yuan Wang et al. [19] proposed a dual-input v-mesh fully convolutional network with the input of raw CT images and algorithmically processed images to improve the contrast between the pancreas and other soft tissues, and enhanced feature extraction by adding an attention module to improve the accuracy of pancreas segmentation. The above methods are able to handle the medical image segmentation problem in an improved way, being combined with FCN [20] or based on the U-Net network [21], and have achieved good segmentation results in respect of different medical images [22]. However, when dealing with the sequence relationship between cardiac MRI images and the multi-scale characteristics of the left atrial structure within the images, they struggle to simulate the continuous relationship between sequential image slices, and cannot capture the contextual information at different scales, thus failing to meet the demand for accurate segmentation.

The GRU network can address the above sequential characteristics of the cardiac MRI image segmentation problem. This network is a classical recurrent neural network that is usually used to learn a set of sequential relationships of data distributed over moment *t*. It can also be used to process a series of 2D images with sequential relationships by converting the temporal parameter *t* into a spatial parameter, with *t* denoting the slice order [23]. In addition, PSPNet [24] utilizes convolutional neural networks with pyramidal pooling to capture target features at different scales [25] and is widely used in image segmentation problems in medicine, agriculture, and geography. The strengths and weaknesses of the common methods in left atrial segmentation are shown in Table 1. Therefore, in this paper, an image segmentation method is proposed based on CNN and RNN. CNN uses a pyramidal pooling network with an attention module to achieve the extraction of targets at different scales in images and to enhance the target features. RNN achieves the acquisition of dependencies between adjacent image slices by designing a bidirectional convolutional GRU network for 2D images. Through this, the final goal of accurate segmentation in the left atrial structure within cardiac magnetic resonance images is achieved.

We summarize the contributions of this paper as follows:

(1) We propose a multiscale feature fusion attention module (MSA), which can capture correlations within spatial dimensions and within channel dimensions using attention mechanisms to enhance target features and improve the network’s ability to distinguish left atrial structures to achieve the capture of targets at different scales in the image.
(2) We design a bidirectional convolutional GRU network, which can simulate the complete anterior-posterior continuous relationship between a set of sequential image slices and use it to obtain the correlation of left atrial structures in adjacent image slices.
(3) We design a combined loss function that allows the network to focus more on the pixel points of the left atrial structure in the image and reduce the effect of positive and negative sample imbalance.

## 2. Cardiac MRI Image Segmentation Problem

The problem of segmenting cardiac magnetic resonance images requires using the patient’s heart magnetic resonance images as input data, classifying all pixels in the image slice to determine whether they belong to the left atrium, and finally segmenting the tissue structure of the left atrium accurately.

**Figure 1:**
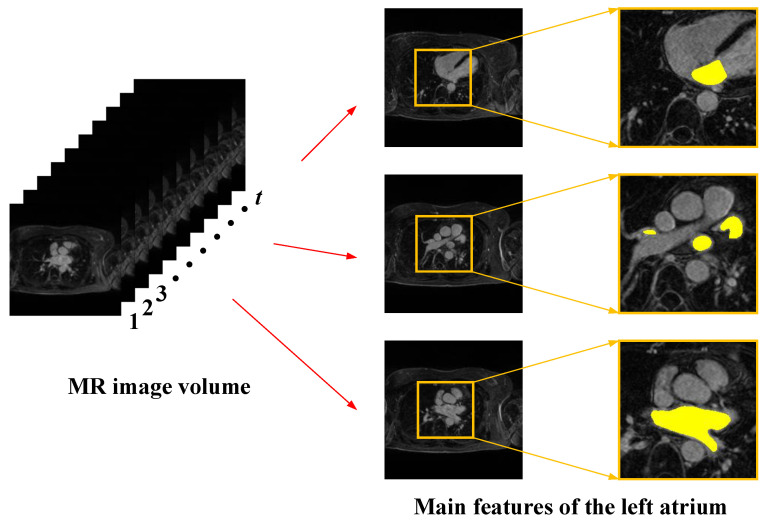
Main features of cardiac magnetic resonance images.

As shown in Figure 1, cardiac MRI images have the following main features:

(1) Cardiac magnetic resonance images have sequential characteristics. Each set of images includes several two-dimensional image slices, and the left atrial structures contained in adjacent slices have continuity, so when extracting the left atrial structure of the current image slice, it is necessary to refer to the relevant information between the before and after image slices.
(2) The left atrial structures in images have multi-scale characteristics. Due to the differences between individual patients and the special characteristics of the left atrial structure, the shape and size of the left atrial structure are not the same in each 2D image slice, and the scale difference is large. Single-scale feature extraction often loses spatial information and affects segmentation accuracy, so the use of multi-scale feature extraction needs to be considered.
(3) The contrast between the left atrium and the surrounding tissue in the image is low. Cardiac MRI images contain not only the left atrial structures but also other structures of the heart, such as the right atrium and the right and left ventricles. These tissue structures have a small difference in pixel values, and the contrast is low, making the tissue structures difficult to distinguish in segmentation.
(4) The percentage of pixel points of the left atrial structures in the images is relatively low. The left atrial pixel points in cardiac MRI images account for less than ten percent of the overall pixel points, and the rest are all background-like pixels. The difference in the number of pixel points between the target and the background is large, which results in a typical positive and negative sample imbalance problem.

## 3. Cardiac MRI Image Segmentation Method

**Figure 2:**
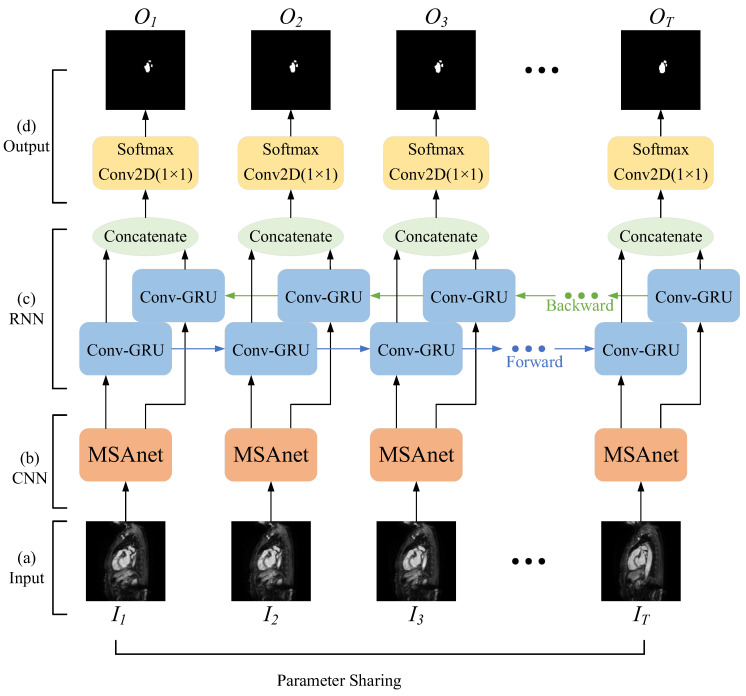
Schematic diagram of the overall network architecture.

The method consists of four main components: an input layer, a convolutional neural network layer with an attention module, a recurrent neural network layer for two-dimensional images, and an output layer. As shown in Figure 2, the overall network structure can be regarded as several two-dimensional semantic segmentation networks with shared parameters in the sequence dimension.

(1) Input layer: Firstly, the original images are split into a set of consecutive 2D images according to the MRI scanning direction; secondly, the image slices that do not contain the left atrial structure are removed and the image size is unified; finally, each set of cardiac MRI images is divided into several image sequences and input into the network.
(2) Convolutional neural network layer with attention module: The features of the left atrial structure are extracted by the convolutional neural network, and then the correlation within the spatial dimension and the channel dimension is captured by the attention mechanism to strengthen the target features and improve the network’s ability to distinguish the left atrial structure to achieve the capture of targets at different scales in the image.
(3) Recurrent neural network layer oriented to two-dimensional images: A bidirectional recurrent neural network with convolutional operations is used to simulate the complete anterior–posterior continuous relationship between a set of sequential image slices, and to obtain the correlation of left atrial structures in adjacent image slices.
(4) Output layer: By constructing a combined loss function, the network is made to focus more on the pixel points of the left atrial structure in the image and reduce the effect of positive and negative sample imbalance. Finally, the class to which each pixel belongs is output to achieve the segmentation of the left atrial structure in cardiac MRI images.

### 3.1. Input Layer

The storage format of medical images is often difficult to apply directly to neural networks, so firstly, the cardiac MRI images were split into a set of consecutive 2D images according to their scanning direction, and the image format was converted to a common computer format. Secondly, due to the MRI equipment and individual patients, there were multiple image slices before and after each set of cardiac MRI images that did not contain the left atrial structure; these image slices without targets were excluded. In addition, the MRI images of different patients had different sizes, and all images were scaled to the same size in order to preserve the details of the training images and reduce the computational complexity of the model.

Each set of MRI images, pre-processed as above, was divided into groups according to the given sequence length T. N was set as the batch-size, so the input size of the network was a five-dimensional tensor of (N,T,C,H,W).

### 3.2. Convolutional Neural Network Layer with Attention Module

#### 3.2.1. U-Shaped Convolutional Neural Network

**Figure 3:**
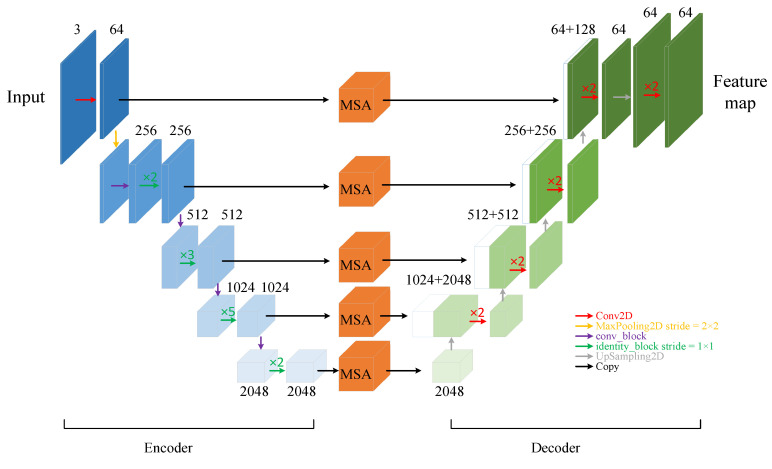
Schematic diagram of convolutional neural network layers with attention module (MSAnet).

The convolutional neural network layer converts the input cardiac MRI image data into a feature matrix, extracts features belonging to the left atrial structure, and then uses the attention mechanism to strengthen these features. The U-Net network, with its excellent performance and high scalability, is widely used for the segmentation of medical images, and its Encoder–Decoder structure, which performs feature fusion by skip connection, effectively improves the image segmentation accuracy. Therefore, as shown in Figure 3, in this study a convolutional neural network layer was designed based on the U-Net network.

Firstly, the backbone feature extraction network is used for the extraction of high-dimensional features. The ResNet network [26] series is the most popular feature extraction network and has a residual structure that introduces the output of a front layer directly into the input part of a later layer by skipping multiple layers, effectively overcoming problems such as the reduced learning efficiency of U-Net networks due to the deepening of the number of network layers. In this study, based on the size of the dataset and the consideration of computing power, the ResNet50 network was preferentially selected as the feature encoder part of the convolutional neural network layers. While gradually extracting high-dimensional features, five groups of feature maps with 64, 256, 512, 1024, and 2048 channels were selected as the initial feature maps to participate in the skip connection part of the fused features.

A multi-scale feature fusion attention module (MSA) was added to the skip connection to capture the left atrial structures with different scales in the image slices and enhance their features. The feature decoder part does not crop the feature maps output by the multi-scale feature fusion attention module, but directly performs a stacking operation with the feature maps obtained by upsampling, which avoids the loss of some information of the left atrial structures in the original U-Net network due to the cropping operation. At the same time, the obtained feature maps are of the same size as the input image, ensuring pixel-level prediction. The final feature map is output by the convolutional neural network layer.

#### 3.2.2. Multi-Scale Feature Fusion Attention Module

To capture the left atrial structures at different scales in the sequence image slices and retain more detailed information [27], an improved dual pyramidal pooling network was added to the multi-scale feature fusion attention module. The input feature map was duplicated and divided into four groups of 1 × 1, 2 × 2, 4 × 4, and 6 × 6 grids, and then a pooling operation was performed on each grid to extract features at different scales in the image through these grids of different sizes. On top of the original average pooling operation, a set of maximum pooling operations of the same type was added to capture the detailed features in the image for the purpose of refining the boundaries of the target region. The eight feature map channels were compressed to one-quarter of the original channel using 1 × 1 convolution, and then upsampling was used to recover the size of the input feature map. The two sets of feature maps obtained by average pooling and maximum pooling were each stacked by channel first, then summed, and finally stacked with the input feature map in the channel dimension to obtain the fused feature map. As a result, the network not only extracts target features at different scales but also enhances the ability to extract image detail information, while reducing the loss of feature information, which helps the subsequent decoder to recover image information by upsampling.

**Figure 4:**
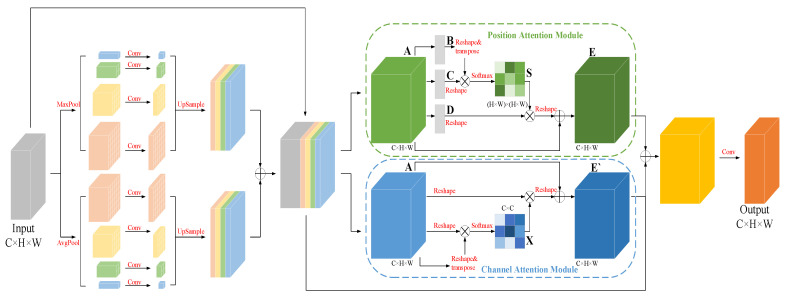
Multi-scale feature fusion attention module (MSA).

In addition, there is a certain topological similarity between different tissues in cardiac MRI images. A double attention mechanism [28] was added in order to exploit the correlations between different regions and enhance the expression of their respective features accordingly. The overall network structure is shown in Figure 4, in which the spatial attention module captures the spatial dependence between any two positions in the feature map by introducing a self-attention mechanism. The feature maps B,C,D are first obtained by the convolution operation, and then the spatial attention map $S\in {\mathbb{R}}^{N\times N}$ is obtained according to the operation shown in Figure 4.

(1) $$s_{ji}=\frac{\exp\left(B_{i}\cdot C_{j}\right)}{\sum_{i=1}^{N}\exp\left(B_{i}\cdot C_{j}\right)}$$

where $B_{i}$ is the $i^{th}$ pixel in feature map B, $C_{j}$ is the $j^{th}$ pixel in feature map C, and $s_{ji}$ measures the influence of the $i^{th}$ position on the $j^{th}$ position in the image. The stronger the association between the two, the larger the value. The final output $E\in {\mathbb{R}}^{C\times H\times W}$ is given by:

(2) $$E_{j}=\alpha \overset{N}{\underset{i=1}{\sum }}\left(s_{ji}D_{i}\right)+A_{j}$$

where the scale parameter α is initialized to 0 and different weights are assigned in the learning. The selective enhancement or suppression of features is achieved by the correlation between pixel points in the global image.

For semantic segmentation, different channels in the feature map can be regarded as responses to different classes, where there is also some correlation. To model this dependency explicitly, a similar self-attentive mechanism is used to capture the correlation between any two channels, and the channel attention graph $X\in {\mathbb{R}}^{C\times C}$ is obtained by the operation shown in Figure 4.

(3) $$x_{ji}=\frac{\exp\left(A_{i}\cdot A_{j}\right)}{\sum_{i=1}^{C}\exp\left(A_{i}\cdot A_{j}\right)}$$

where $A_{i}$ and $A_{j}$ denote the $i^{th}$ and $j^{th}$ channels in the feature map A, and $x_{ji}$ denotes the influence of the $i^{th}$ channel on the $j^{th}$ channel in the feature map A. The final output $E‘\in {\mathbb{R}}^{C\times H\times W}$ is given by:

(4) $$E{‘}_{j}=\beta \overset{C}{\underset{i=1}{\sum }}\left(x_{ji}A_{i}\right)+A_{j}$$

where the scale parameter β is initialized to 0 and different weights are assigned in the learning, in order to enhance the responsiveness of specific semantics under a channel by simulating the correlation between channels.

Through the dual pyramidal pooling network and dual attention mechanism in the multi-scale feature fusion attention module, the network can effectively capture the left atrial structure at different scales and enhance the representation of left atrial features.

### 3.3. Recurrent Neural Network Layer Oriented to Two-Dimensional Images

#### 3.3.1. Convolutional GRU Network

**Figure 5:**
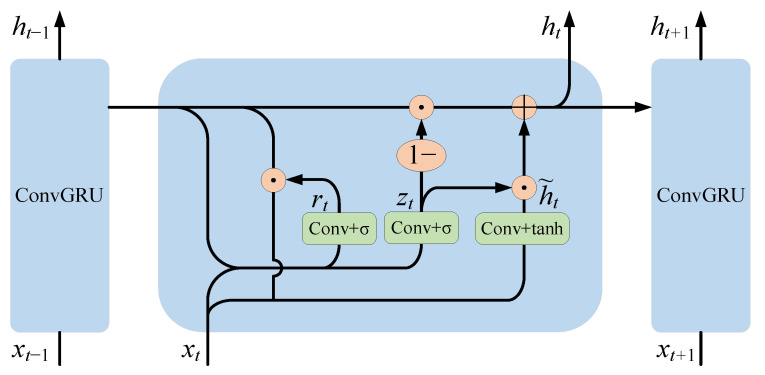
The internal principle of the convolutional GRU cell.

In order to simulate the continuous relationship between slices in cardiac MRI images, a recurrent neural network layer was designed for two-dimensional images. The GRU network [29], as a classical recurrent neural network, achieves sequence information association by retaining previous feature information and has the advantages of a simple structure and easy computation and training. Unlike a traditional recurrent neural network, which is only applicable to one-dimensional sequence data, the convolutional GRU (ConvGRU) network is capable of processing cardiac MRI sequence images by replacing the original fully connected operation in the GRU network with a convolutional operation. The internal principle of the convolutional GRU unit is shown in Figure 5.

ConvGRU [30] is defined by the following equations:

(5) $$z_{t}=\sigma \left(W_{z}*x_{t}+U_{z}*h_{t-1}+b_{z}\right)$$

(6) $$r_{t}=\sigma \left(W_{r}*x_{t}+U_{r}*h_{t-1}+b_{r}\right)$$

(7) $${\tilde{h}}_{t}=tanh\left(W*x_{t}+U*\left(r_{t}⊙h_{t-1}\right)+b\right)$$

(8) $$h_{t}=\left(1-z_{t}\right)⊙h_{t-1}+z_{t}⊙{\tilde{h}}_{t}$$

where ∗ denotes the convolution operation, ⊙ denotes element-wise multiplication, $x_{t}$ is the input at moment t, W and U are trainable network parameters, and b is the bias term. ${\tilde{h}}_{t}$ denotes the candidate hidden state. $z_{t}$ denotes the update gate. $r_{t}$ denotes the reset gate. $h_{t}$ denotes the pixel-level feature map output at moment t, which is consistent in spatial size with the input features.

Through the simulation of the sequence relationship, the deep semantic features of the left atrial structure in the current image slice can be extracted and retained in the network until the next image slice is segmented, which can effectively improve the segmentation accuracy.

#### 3.3.2. Bidirectional Convolutional GRU Network

The convolutional GRU network described above is a one-way structure, where the current output is determined by the previously learned information and the current input. Since the left atrial structures between adjacent slices in cardiac MRI images have continuity, the forward and backward sequences have the same importance [31], and only modeling unidirectional sequence relationships will lead to compromised segmentation accuracy.

The bidirectional convolutional GRU (Bi-ConvGRU) network uses a combination of two layers of convolutional GRU networks in opposite directions. The network provides a comprehensive view of the entire sequence of cardiac MRI images, simulates the correlation between the forward and backward images, and deeply mines the correlations of left atrial structures between adjacent slices.

**Figure 6:**
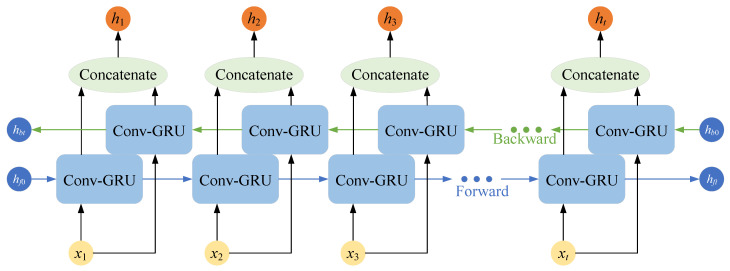
Schematic diagram of the structure of the Bi-ConvGRU network.

The bidirectional convolutional GRU network shown in Figure 6 outputs two sets of feature maps, forward and backward, which help the network to capture the correlations of left atrial structures in adjacent slices by stacking these two sets of feature maps and thus fusing the complete sequence information of cardiac MRI images. The recurrent neural network layer oriented to 2D images finally outputs feature maps of size (2*C*,*H*,*W*).

### 3.4. Output Layer

The output layer implements the class prediction for each pixel point in the cardiac MRI image based on the image features extracted and fused in the previous two stages. The segmentation task in this paper was essentially the binary classification of each pixel point within the image, so 1 × 1 convolution was used to first adjust the feature map channels of the output from the recurrent neural network layer to the number of classes, and then the Softmax function was used as the activation function to obtain the predicted probability map of the input image.

For the low percentage of pixel points in the left atrial structure among the images, a combined loss function was used to alleviate this positive and negative sample imbalance. The loss function was first designed based on the Dice similarity coefficient (DSC).

(9) $$Loss_{dice}=1-\frac{2*\left|H_{p}\cap H_{g}\right|}{\left|H_{p}\right|+\left|H_{g}\right|}$$

where $H_{p}$ is the predicted segmentation of the network and $H_{g}$ is the labeled image.

Focal Loss [32] was added to Dice Loss, resulting in a dynamic scaling cross-entropy loss based on binary cross-entropy. With the dynamic scaling factor, the weight of the easily distinguishable samples can be automatically reduced during the training process, so that the emphasis can be quickly focused on those samples that are difficult to distinguish, allowing the network to better classify each pixel point. Focal Loss is defined by the following equation:

(10) $$Loss_{focal}=-\alpha y{\left(1-\hat{y}\right)}^{\gamma }log\hat{y}-\left(1-\alpha \right)\left(1-y\right){\hat{y}}^{\gamma }log\left(1-\hat{y}\right)$$

where y is the actual category. $\hat{y}$ is the predicted category of the classifier. The parameter α is used to adjust the ratio between positive and negative samples. γ is a positive adjustable parameter that automatically adjusts the loss contribution of positive and negative samples.

Therefore, the final loss function in this paper was:

(11) $$Loss_{seg}=Loss_{dice}+Loss_{focal}$$

The above prediction method and loss function were designed so that the network pays more attention to fewer samples while predicting each pixel in the image, which effectively improves the accuracy of the model prediction segmentation.

## 4. Experimental Results and Analysis

### 4.1. Experimental Dataset

In this paper, the proposed network model was evaluated using two left atrial segmentation datasets, one of which was the Left Atrial Segmentation Challenge dataset (2013) [8], and the other was the Atrial Segmentation Challenge dataset (2018) [33]. The different scan orientations and different data sizes of the MRI images in the two datasets enabled effective evaluation of the generalization of the network.

#### 4.1.1. Left Atrial Segmentation Challenge Dataset (LASC2013)

The dataset provided by STACOM’13 in MICCAI’13 is a small dataset containing a total of 30 MRI images; 10 MRI images were used as the training set with manually segmented labeled images, and the rest were used as the test set without manually segmented labels. Therefore, only 10 of these images with true labels were utilized as a dataset in this paper. Each sample included the left atrium and its appendages (LAA) with 100–120 slices, of which a large number of slices do not include segmentation targets and were excluded. The actual number of slices used in the experiment was 608. The small sample size and the changes in scale presented a challenge for the accurate segmentation of the left atrium.

#### 4.1.2. Atrial Segmentation Challenge Dataset (ASC2018)

This dataset is from the MICCAI 2018 Atrial Segmentation Challenge and is a large medical clinical dataset that includes MRI images of 154 patients with AF. The raw resolution of the data is 0.625 × 0.625 × 0.625 mm^3^, with sizes ranging from 576 × 576 to 640 × 640 pixels. Each patient’s MRI image data contains both the raw data and the corresponding left atrial labels manually labeled by experts in the field. The raw data are grayscale maps and the segmentation labels are binary maps (255 = positive, 0 = negative), in which white represents the target region and black represents the background region. Each patient has different-sized MRI images due to individual disparities, but the images all contain 88 slices in the *Z*-axis. The left atrial region of this dataset is small and the target size is highly variable, which poses a difficult problem in semantic segmentation.

### 4.2. Experimental Setup

The proposed medical image segmentation method, based on sequence relationship learning and multi-scale feature fusion, was implemented with Python and the Pytorch deep learning framework, and the program was run on a server with an Nvidia Tesla V100 GPU and Unbuntu16.04 operating system.

In this study, in order to preserve the training image details and reduce the computational complexity of the model, the images of the datasets were scaled to a size of 512 × 512 pixels (640 × 640 pixels on ASC2018), and the length of each time sequence was set to eight when training the network. Stochastic gradient descent (SGD) was used as the optimizer for the network training, and the training was divided into three steps. In the first step, the convolutional neural network layer with an attention module was first trained for the segmentation of static images with 120 iterations and a batch size of 16 subjects. The initial learning rate was 0.01, and it decayed after every 10 rounds of training. In the second step, the complete network of this paper was constructed, and the weights of the convolutional neural network layer obtained by pre-training were loaded and frozen so that the weights were not involved in updating during training; only the recurrent neural network layer was trained until convergence. The learning rate was set as in the previous step. In the third step, the weights of the convolutional neural network layer were unfrozen, and a smaller learning rate was set, in which the whole network was trained jointly until convergence. In the prediction process, the prediction results of the network were binarized to obtain the predicted segmented image.

The focus of this study was the binary classification problem, and a total of three evaluation metrics, the Dice coefficient [34], intersection over union (IoU), and Hausdorff distance [35], were selected to evaluate the segmentation results.

(12) $$ice=\frac{2\times \left|H_{p}\cap H_{g}\right|}{\left|H_{p}\right|+\left|H_{g}\right|}\times 100\%$$

(13) $$IoU=\frac{\left|H_{p}\cap H_{g}\right|}{\left|H_{p}\cup H_{g}\right|}\times 100\%$$

where $H_{p}$ is the predicted segmentation of the network; $H_{g}$ is the labeled image. The Dice coefficient is one of the most commonly used evaluation metrics in medical image segmentation, and the range of values is generally (0~1).

The Hausdorff distance is the surface distance that measures the maximum distance from the point sets to the nearest point in other sets.

(14) $$D_{H}\left(X,Y\right)=max\left\{sup_{x\in X}inf_{y\in Y}d\left(x,y\right),sup_{y\in Y}inf_{x\in X}d\left(x,y\right)\right\}$$

where *sup* is the least upper bound; *inf* is the greatest lower bound; *d* is the Euclidean matrix.

By using the above-mentioned evaluation metrics, the segmentation results of the model can be measured from multiple perspectives, which makes the final evaluation more objective and comprehensive.

### 4.3. Experimental Results

#### 4.3.1. Comparison Experiment between the Proposed Method and a Traditional Network

**Figure 7:**
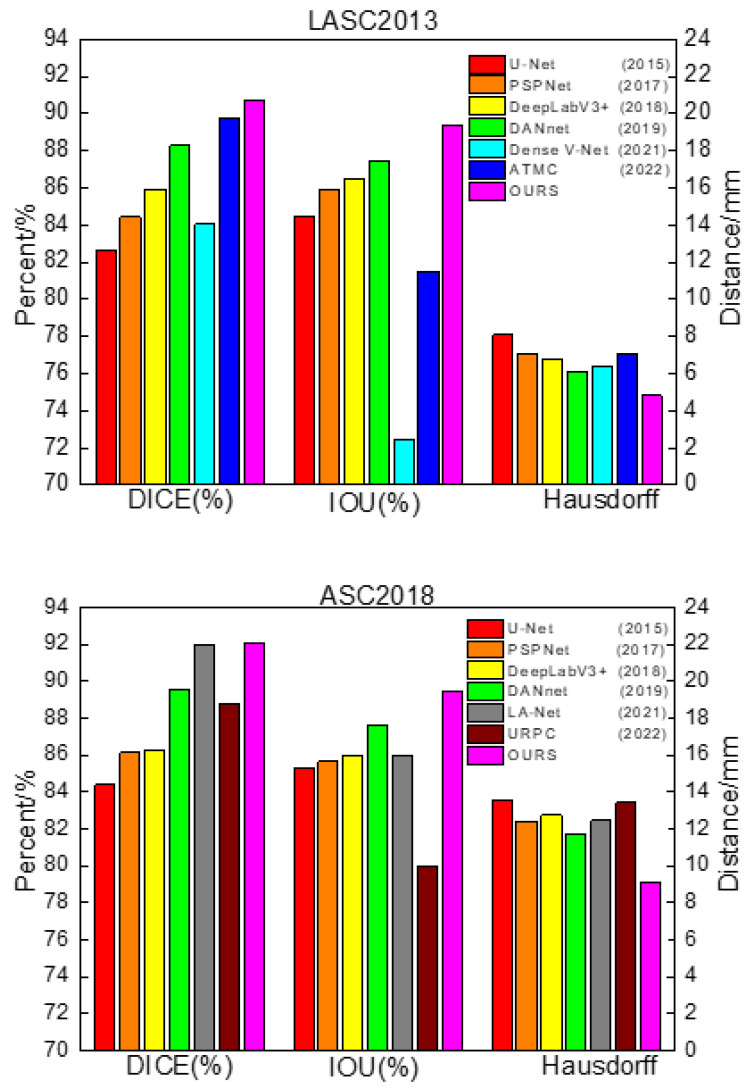
Comparison of segmentation performance of different networks.

In order to verify the effectiveness of the network proposed in this paper, traditional networks, U-Net, DeepLabV3+ [36], PSPNet, and DANet were selected for comparison. During training, the image input size of each model was uniformly adjusted to 512 × 512 pixels (640 × 640 pixels on ASC2018) to reduce the influence of parameter settings on the segmentation performance between different networks. The batch size was set to 16, the initial learning rate was 0.01, the minimum learning rate was 0.0001, and the cosine annealing [37] was used to decay the learning rate. The experiments were divided into training and validation sets according to the ratio of 4:1. In addition, the latest methods that achieved excellent performance in the LASC2013 and ASC2018 datasets were selected for comparison [38,39,40,41,42]. The segmentation performance of the different models is shown in Table 2 and Figure 7.

From Table 2, it can be concluded that U-Net, as a baseline for general semantic segmentation that fuses different kinds of depth information by skipping connections, still achieved good results. PSPNet uses the pyramid pooling module to aggregate contextual information at different scales, giving it better global information extraction capability, but it only utilizes deep features and fails to fuse shallow features, which leads to the loss of some detailed information, so the segmentation effect at the left atrial border was poor. DeepLabV3+ uses a more efficient feature extractor with better control of boundary information and adopts atrous spatial pyramid pools for multi-scale feature extraction, helping it achieve good segmentation results. DANet strengthens the target features based on the self-attention mechanism and extracts the relationship between different objects from a global perspective, but it also failed to integrate the shallow information, easily lost the image details, and had a poor segmentation effect at the boundary.

The above methods achieved good segmentation results, but their common drawback was that they did not highlight the characteristics of cardiac MRI images with sequence relationships and low contrast between the left atrium and the surrounding tissues. The proposed method in this paper uses multi-scale feature fusion to improve the processing of global and detailed images based on skipping to connect shallow features and deep features and uses recurrent neural network layers to simulate the sequence relationships of cardiac MRI images, thus achieving better segmentation results in the left atrial dataset.

**Figure 8:**
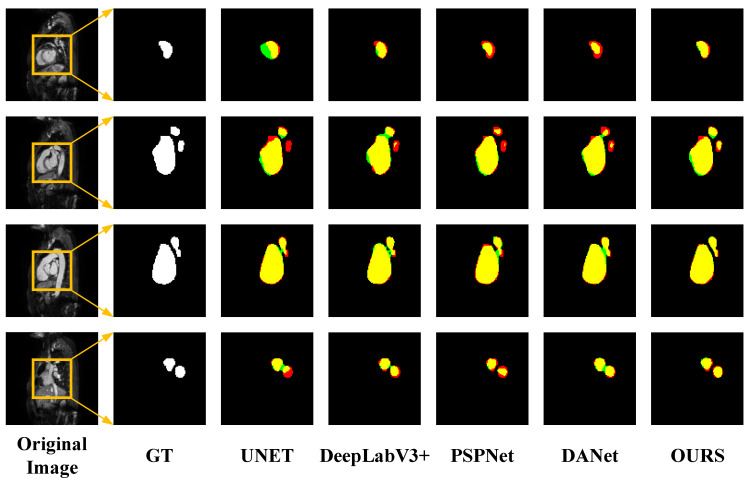
Segmentation results for different networks on the LASC2013 dataset.

**Figure 9:**
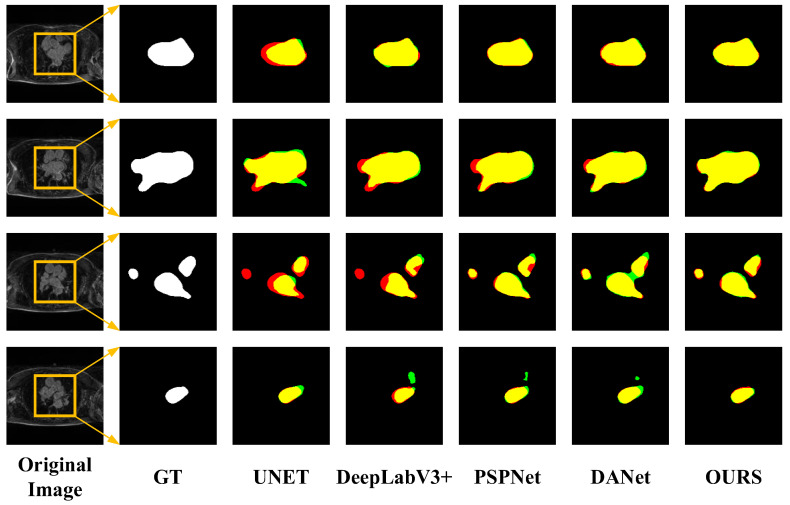
Segmentation results for different networks on the ASC2018 dataset.

However, from Table 3, it can be concluded that the performance of the proposed method in LASC 2013 on the Dice metric is not as superior as the methods provided in the challenge. Although we have not achieved top performance, the proposed method does not require structural changes or additional processing to achieve relatively good performance and has some versatility and convenience to meet certain clinical needs. The advantages of the traditional method can be learned in the next study and applied to the proposed method to further improve the performance. In order to visually compare the segmentation effects of various methods, some of the predicted segmentation images from one example in each of the two datasets are shown below for comparison with the real segmentation images. Among them, the first column shows the MRI images of the heart to be segmented. The second column shows the left atrial structures manually labeled by the expert. Starting from the third column are the segmentation results for different models. The labeled images are represented in red, the model-predicted segmentation images are represented in green, and the overlap region between the two is represented in yellow. As shown in Figure 8 and Figure 9 below, the existing methods achieved good segmentation results on target slices with smooth boundaries and high contrast, but sequence relationships and multi-scale feature fusion were highlighted in this study, so it also maintained good segmentation results on small-scale targets and target slices with low contrast.

#### 4.3.2. Network Validity Experiment

Ablation experiments were designed to verify the effectiveness of the convolutional neural network layer and recurrent neural network layer proposed in this paper and were set up as follows: (1) the backbone feature extraction module was replaced by ResNet50 on the basis of U-Net and was named RUNet in the experiments. (2) On the basis of RUNet, a multi-scale feature fusion attention module was constructed, which was named MUNet in the experiment. (3) On the basis of RUNet, a bidirectional convolutional GRU network was constructed, which was named SeqUNet in the experiment. (4) The complete network proposed in this paper. The model training settings in the ablation experiments were the same as those in the previous comparison experiments. The experimental results are shown in Table 4.

It can be inferred from Table 4 that RUNet improved the segmentation compared with the original U-Net, indicating that the residual structure in the replaced feature extractor can effectively improve the segmentation accuracy. In addition, MUNet and SeqUNet had different degrees of segmentation accuracy improvement, indicating that the use of a convolutional neural network layer with attention and a recurrent neural network layer oriented to two-dimensional images can effectively improve the segmentation accuracy. The method presented in this paper combines the advantages of the above two structures so that the best segmentation accuracy was achieved and the effectiveness of the method for improving the network segmentation accuracy was verified.

## 5. Conclusions

In this paper, an image segmentation method based on sequence relationship learning and multi-scale feature fusion was proposed for 3D to 2D sequence conversion in cardiac magnetic resonance images and of the left atrial structure with different scales within different image slices. Firstly, the method automatically extracted target features at different scales and performed deep fusion through the design of a convolutional neural network layer with attention. This enhanced the capture of detailed information based on the acquisition of global features by using the correlation between different regions within the image and refined the target region boundaries. Secondly, for the sequence characteristics of cardiac MRI images, a recurrent neural network layer oriented to two-dimensional images was constructed to simulate the continuous relationship between sequence images and capture the correlation of left atrial structures between adjacent slices. Finally, a combined loss function was used to solve the positive and negative sample imbalance problem to a certain extent. In segmentation experiments based on the LASC2013 and ASC2018 left atrial datasets, the Dice scores of the proposed method in this paper were 90.73% and 92.05%, respectively, confirming better segmentation accuracy compared to the traditional network.

The results demonstrated that the proposed method could obtain good results under most situations. However, some previous traditional methods perform better results in some metrics. In our future research work, we will learn the advantages of the traditional methods to make up for our own shortcomings. In addition, the semi-supervised or even unsupervised methods can be considered to reduce the reliance on labeled data, and the segmentation accuracy of the proposed method can be further improved by the above methods.

## Figures and Tables

**Table 1:** Methods for left atrial image segmentation.

Methods	Strengths	Weaknesses
Traditional algorithms [5,6,7]	Achieve good results for specific problems	Require manual feature extraction
Yashu Liu et al. [12]	Reduce the effect of imbalance between positive and negative samples	Does not consider sequence relationships and low accuracy
Zhaohan Xiong et al. [13]	Extract multi-scale feature	Does not consider Sequence relationships
Sulaiman Vesal et al. [14]	Exploited Sequential relationships through 3D convolution	Require large datasets and difficult to capture long-range dependencies
OUR	Extract multi-scale features and capture sequence relationship	Require complex structure and high calculation volume

**Table 2:** Comparison of segmentation performance of different networks.

Dataset	Network	DICE (%)	IOU (%)	Hausdorff Distance (mm)
LASC2013	U-Net (2015) [21]	82.67	84.47	8.068
	PSPNet (2017) [24]	84.42	85.95	7.049
	DeepLabV3+ (2018) [36]	85.93	86.48	6.819
	DANet (2019) [28]	88.27	87.46	6.118
	Dense V-Net (2021) [38]	84.03	72.46	6.430
	ATMC (2022) [39]	89.75	81.51	7.020
	OURS	90.73	89.37	4.803
ASC2018	U-Net (2015) [21]	84.38	85.33	13.533
	PSPNet (2017) [24]	86.11	85.64	12.385
	DeepLabV3+ (2018) [36]	86.25	85.98	12.722
	DANet (2019) [28]	89.51	87.59	11.709
	LA-Net (2021) [40]	92.00	86.00	12.430
	URPC (2021) [41,42]	88.74	79.93	13.400
	OURS	92.05	89.41	9.056

**Table 3:** Performance comparison with traditional methods in LASC2013.

Methods	DICE (%)
Zuluaga et al. (2013) [43]	92.00
Stender et al. (2014) [44]	87.06
Sandoval et al. (2014) [45]	94.20
OURS	90.73

**Table 4:** Performance comparison among different network architectures.

Dataset	Network	DICE (%)	IOU (%)	Hausdorff Distance (mm)
LASC2013	RUNet	83.49	85.43	7.767
	MUNet	88.44	88.04	5.701
	SeqUNet	87.48	87.44	5.098
	OURS	90.73	89.37	4.803
ASC2018	RUNet	86.45	85.84	10.811
	MUNet	89.13	87.56	10.124
	SeqUNet	88.69	87.42	10.719
	OURS	92.05	89.41	9.056

## Data Availability

Not applicable.
